# Resistance‐guided isolation and characterization of antibiotic‐producing bacteria from river sediments

**DOI:** 10.1186/s12866-021-02175-5

**Published:** 2021-04-17

**Authors:** Nowreen Arefa, Ashish Kumar Sarker, Md. Ajijur Rahman

**Affiliations:** 1 Department of Pharmacy, University of Rajshahi, Rajshahi, Bangladesh; 2 Department of Pharmacy, Pabna University of Science and Technology, Pabna, Bangladesh

**Keywords:** Antibiotic resistance, *Streptomyces*, Antibiotic-resistant isolates, 16S rDNA sequencing

## Abstract

**Background:**

To tackle the problem of antibiotic resistance, an extensive search for novel antibiotics is one of the top research priorities. Around 60% of the antibiotics used today were obtained from the genus *Streptomyces*. The river sediments of Bangladesh are still an unexplored source for antibiotic-producing bacteria (APB). This study aimed to isolate novel APB from Padma and Kapotakkho river sediments having the potential to produce antibacterial compounds with known scaffolds by manipulating their self-protection mechanisms.

**Results:**

The antibiotic supplemented starch-casein-nitrate agar (SCNA) media were used to isolate antibiotic-resistant APB from the river sediments. The colonies having Streptomyces-like morphology were selectively purified and their antagonistic activity was screened against a range of test bacteria using the cross-streaking method. A notable decrease of the colony-forming units (CFUs) in the antibiotic supplemented SCNA plates compared to control plates (where added antibiotics were absent) was observed. A total of three azithromycin resistant (AZR) and nine meropenem resistant (MPR) isolates were purified and their antagonistic activity was investigated against a series of test bacteria including *Shigella brodie*, *Escherichia coli*, *Pseudomonas sp.*, *Proteus sp., Staphylococcus aureus*, and *Bacillus cereus*. All the AZR isolates and all but two MPR isolates exhibited moderate to high broad-spectrum activity. Among the isolates, 16S rDNA sequencing of NAr5 and NAr6 were performed to identify them up to species level. The analyses of the sequences revealed that both belong to the genus *Streptomyces*.

**Conclusions:**

The results from these studies suggest that manipulation of the self-resistance property of APB is an easy and quick method to search for novel APB having the potential to produce potentially novel antibacterial compounds with known scaffolds.

## Background

Although the first natural antibiotic, penicillin, was discovered from a mould, *Penicillium notatum*, majority of the other clinically useful antibiotics including chloramphenicol, tetracycline, erythromycin, gentamycin, neomycin, streptomycin, etc. were obtained from a group of soil-dwelling filamentous gram-positive bacteria known as actinomycetes, particularly from the genus *Streptomyces*. About 70% of the antibiotics that have been approved to use in humans are the secondary metabolites produced by actinomycetes [1, 2]. However, the use of the traditional approach for screening of actinomycetes, known as the Waksman platform, to search for novel antibiotics was abandoned as this approach often resulted in the rediscovery of known compounds [3]. Moreover, the failure of the pharmaceutical companies to identify novel scaffolds by screening the synthetic libraries and by using rational drug design led most companies to abandon antibiotic discovery research [3]. Using the traditional screening approaches, several millions of actinomycetes colonies need to be screened to discover new antimicrobial compounds with known scaffolds [4]. For instance, to find a glycopeptides antibiotic (GPA) producer, an estimated number of 150,000 strains of actinomycetes need to be screened [4, 5]. However, using the resistance-based screening, a GPA-producing strain can be identified by screening only 10 strains [4].

Moreover, exploration of the relatively untapped sources of antibiotic-producing bacteria (APB) especially the marine and extreme environments [6, 7]; screening the unculturable soil bacteria [8] as well as human microbiota [9] have resulted in the discovery of novel antimicrobials. For instance, using the isolation chip (iChip), teixobactin, a new class of antibiotics with novel scaffolds, was discovered from *Eleftheria terrae*, an unculturable soil bacteria [8]. Another new class of antibiotics, lugdunin, was discovered from *Staphylococcus lugdunensis*, a gram-positive bacterium colonising the human nasal cavity [9]. Pekiskomycin, a novel glycopeptide antibiotic (GPA) was discovered from *Streptomyces sp.* WAC4229 by selecting the GPA-producing organisms using vancomycin [4]. These recent success stories on the discovery of novel antimicrobial compounds suggest that if the relatively untapped sources are explored with novel strategies, there is still a chance of obtaining new antibiotics with novel scaffolds.

The sediments deposited at the riverbed consist of sand, soil, rocks, minerals as well as residues of plants and animals. The river sediments also contain a large number of bacteria, predominantly *Proteobacteria* [10]. Although there are reports on the isolation of actinomycetes producing antibacterial compounds from river sediments [11–14], according to our knowledge, there are no studies on the isolation of APB from the river sediments of Bangladeshi rivers, thus, still, this source has remained untapped. Here, in this study, we have reported the isolation of actinomycetes from the river sediments of Bangladesh using the antibiotic resistance-guided approach. The results of antibiotic supplementation (azithromycin or meropenem) in the actinomycete isolation medium, the antibacterial activity of the antibiotic-resistant isolates as well as the identification of some selective bioactive isolates are also presented.

## Results

### Antibiotic supplementation in the SCNA media reduced the load of colonies on the isolation plates

**Fig. 1:**
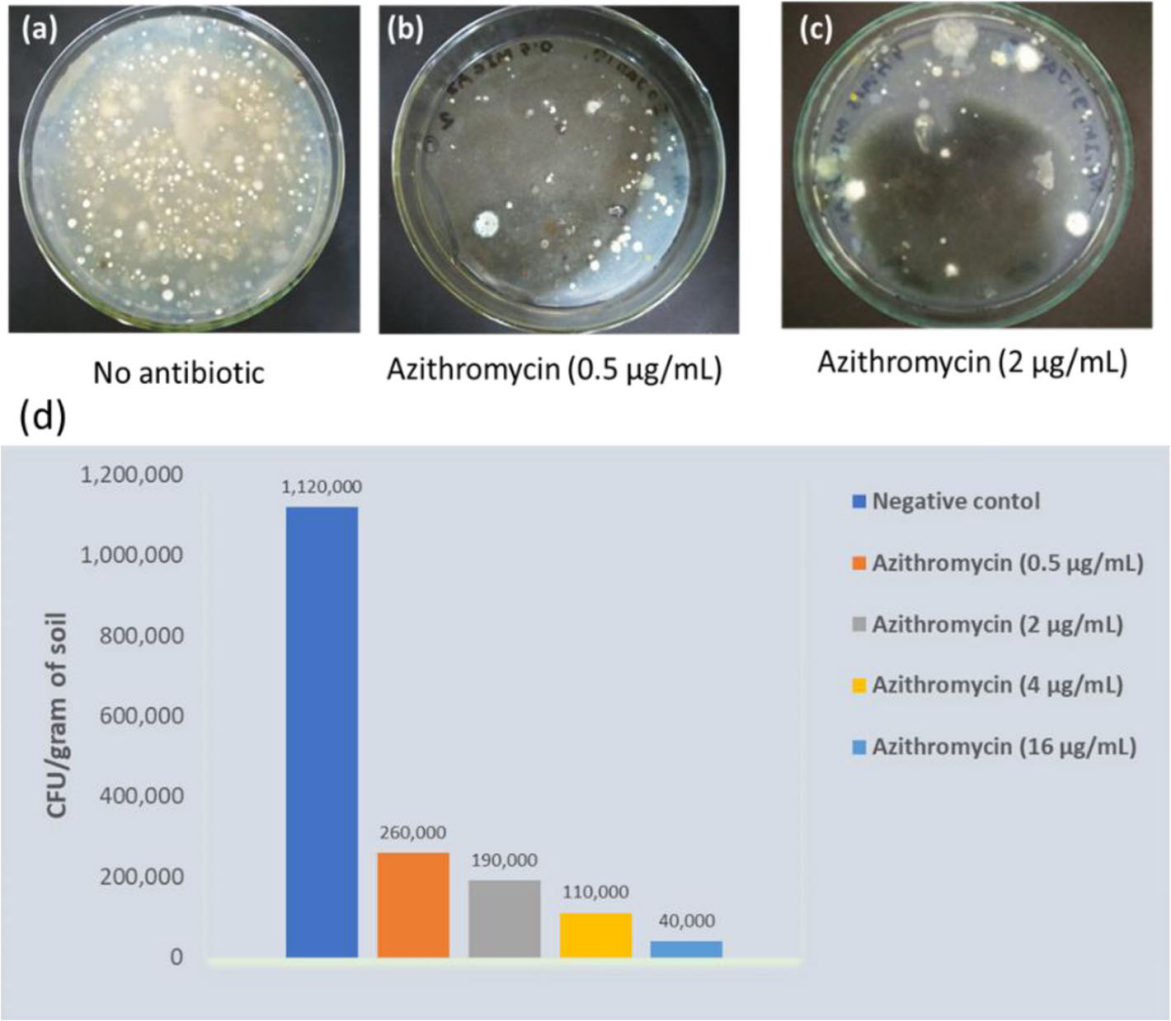
Colonies appeared on the SCNA plates with or without supplementation of azithromycin by plating 100 µL of the river sediments of 1: 100 dilution. **a**, (**b**) and (**c**); actinomycete colonies appeared on the control SCNA plates, plates where 0.5 µg/mL of azithromycin was added and 2 µg/mL azithromycin was added, respectively.  The number of CFU/gram of soil of when the suspension of 1:100 dilution was plated on to the SCNA plates supplemented with different concentration of azithromycin

In this study, two antibiotics namely, azithromycin and meropenem were used separately to isolate drug-resistant actinomycetes with potential of producing antibiotics to which they possess self-resistance mechanisms. It was found that the antibiotic supplementation killed the susceptible bacterial species present in the sample and reduced the number of bacteria on the isolation plates by 4–6 folds compared to the control plates where no antibiotics were added. This allowed only the drug-resistant actinomycetes to grow, especially the actinomycetes which are difficult to grow on the crowded plates. For instance, in SCNA plate containing 0.5 µg/mL azithromycin where 100 µL of sediment sample (1:100 dilution) of Padma river was plated and the number of CFU/gram of soil was counted after 4 days of incubation at 32 °C, the number of CFU/gram of soil was 2.6 × 10^5^, however, in the control SCNA plates where no antibiotic was added, the CFU/gram of soil was 1.12 × 10^6^ (Fig. 1). When the antibiotic concentration in the SCNA plates was increased, the number of CFU/gram of soil also decreased proportionately. For example, the CFU/gram of soil in the plates supplemented with 2, 4 and 16 µg/mL of azithromycin, the values for CFU/gram of soil were 1.9 × 10^5^, 1.1 × 10^5^ and 4 × 10^4^, respectively.

**Fig. 2:**
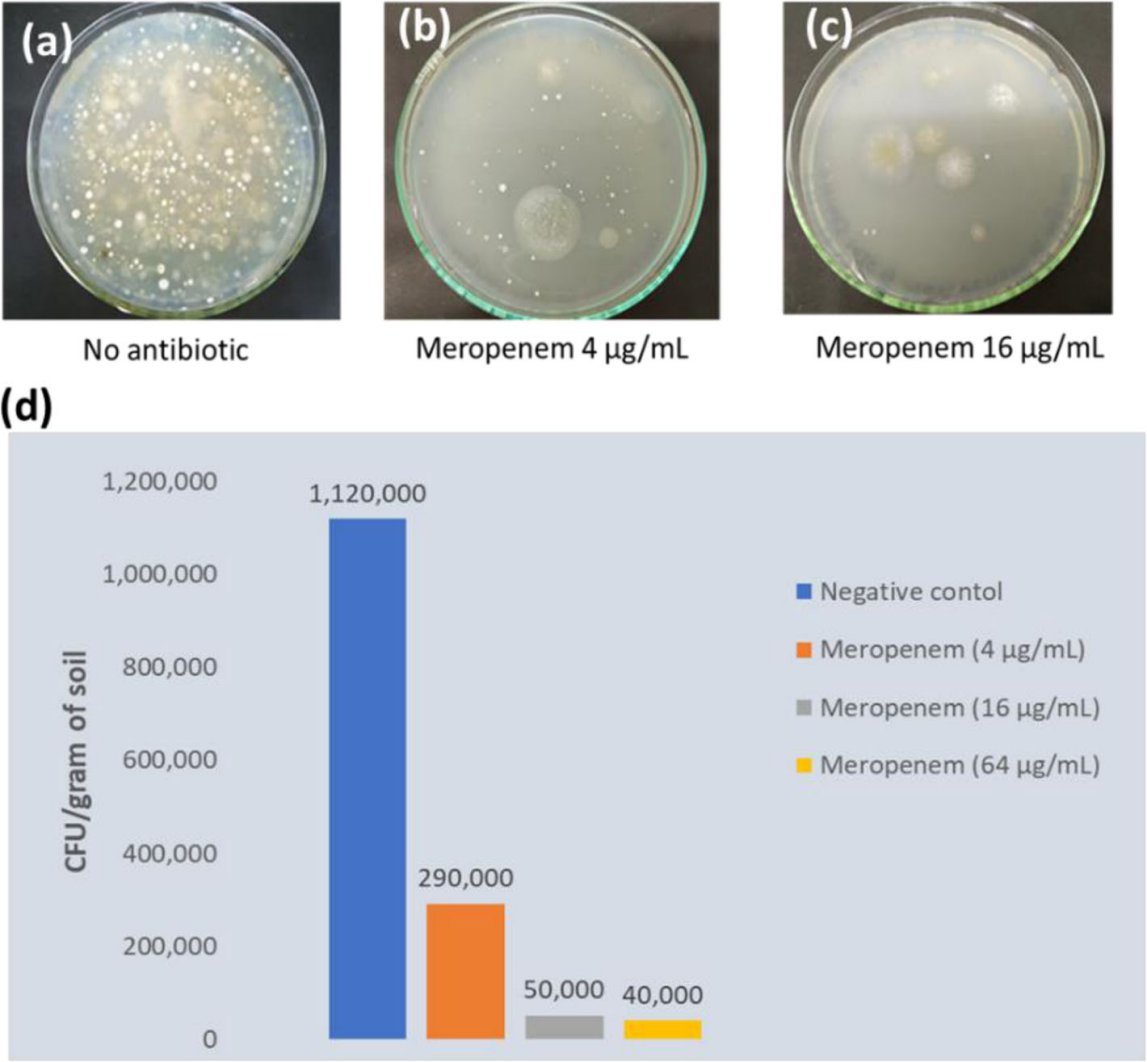
Colonies appeared on the SCNA plates with or without supplementation of meropenem by plating 100 µL of the river sediments of 1: 100 dilution. (**a**), (**b**) and (**c**); actinomycete colonies appeared on the control SCNA plates, plates where 4 µg/mL and 4 µg/mL of meropenem was added, respectively. **d** The number of CFU/gram of soil of when the suspension of 1:100 dilution was plated on to the SCNA plates supplemented with different concentration of meropenem

Similarly, when the SCNA plates were supplemented with meropenem, at least a four-fold decrease in the number of meropenem-sensitive soil bacteria appeared on the isolation plates was observed (Fig. 2). For instance, on the plating of the 1:100 diluted river sediments of Padma river on SCNA plates supplemented with 4 µg/mL, the number of CFU/gram of sediment was counted as 2.9 × 10^5^, whereas on the control plates where the number of CFU/gram of sediment was 1.12 × 10^6^ which is around four-fold higher than the meropenem supplemented plates (Fig. 2).

### Pure culture of the antibiotic resistance isolates exhibited morphological characteristics similar to actinomycetes, particularly *Streptomyces*

**Fig. 3:**
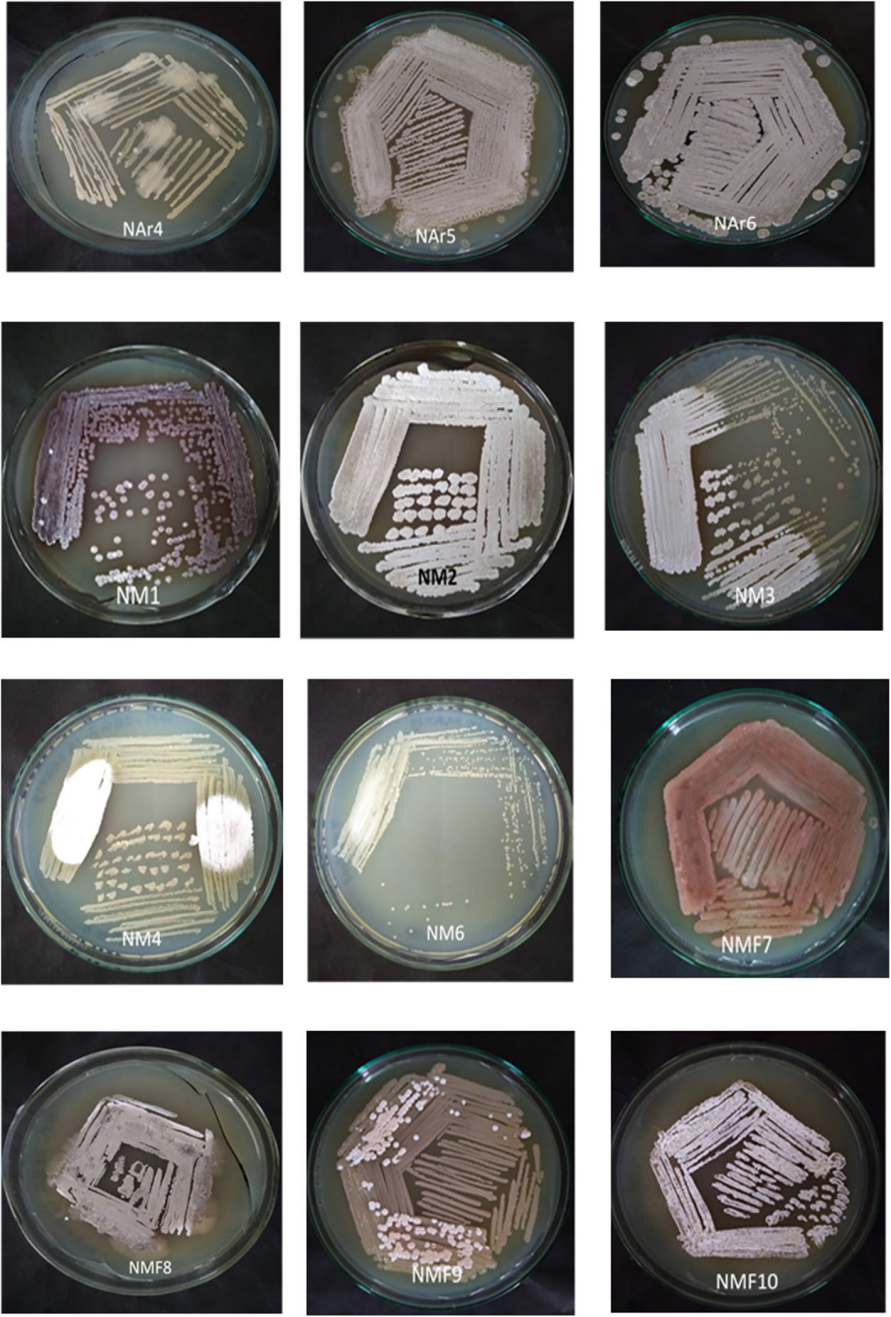
The pure isolates of actinomycetes strains isolated from river sediments using resistance-guided isolation procedure. The isolates were grown on YEGA plates (supplemented with either azithromycin or meropenem) for 4 days at 32 °C

A total of 12 antibiotic-resistant actinomycete isolates were purified. Among them, three were from azithromycin supplemented plates and nine were from meropenem supplemented plates (Table 1). Most of the isolates represented the morphological characteristics of *Streptomyces* as they showed good sporulation with compact, chalk-like colonies of different colours. The separately appeared colonies having the morphology of actinomycetes especially the *Streptomyces* were picked randomly from the SCNA plates and inoculated on the fresh yeast-extract glucose agar (YEGA) plates supplemented with the same concentration of antibiotics. To confirm that the isolates were pure and no contaminant bacteria or fungi were present, the isolates were transferred to fresh plates for at least two times. By analysing the aerial mycelial views of the pure isolates (Fig. 3.), they were classified into different colour groups. The white and grey-coloured isolates were predominant. All the isolates obtained from azithromycin-supplemented plates were grouped into grey series. Among the nine isolates purified from meropenem supplemented plates, six were grouped to white series (NM1, NM2, NM3, NM4, NM6 and NMF10) and two were grouped to grey series (NMF8 and NMF9). The isolate NMF7 was grouped into red series. Only one isolate (NM1) was found to produce red-coloured diffusible pigments into the media.

**Table 1:** Collection site and isolation plates from where azithromycin and meropenem resistant isolates were collected

Date of collection	Collection site of the river sediments	Depth	Antibiotic supplemented on the SCNA plates	Concentration of the antibiotics on the plates	Isolate
30/01/19	Kopotakkha, Jashore (23.1061737 N,89.0937548E)	Approx. 2–3 m	Azithromycin	0.5 µg/mL	NAr4, NAr5
				4 µg/mL	NAr6
03/03/19	Padma, Rajshahi (24.3614252 N,88.5991637E)	Approx. 2–3 m	Meropenem	4 µg/mL	NM1, NM2, NM3, NM4, NM6, NMF7, NMF8, NMF9, NMF10

### Most of the pure isolates obtained from azithromycin and meropenem selection were active against the test bacteria

**Fig. 4:**
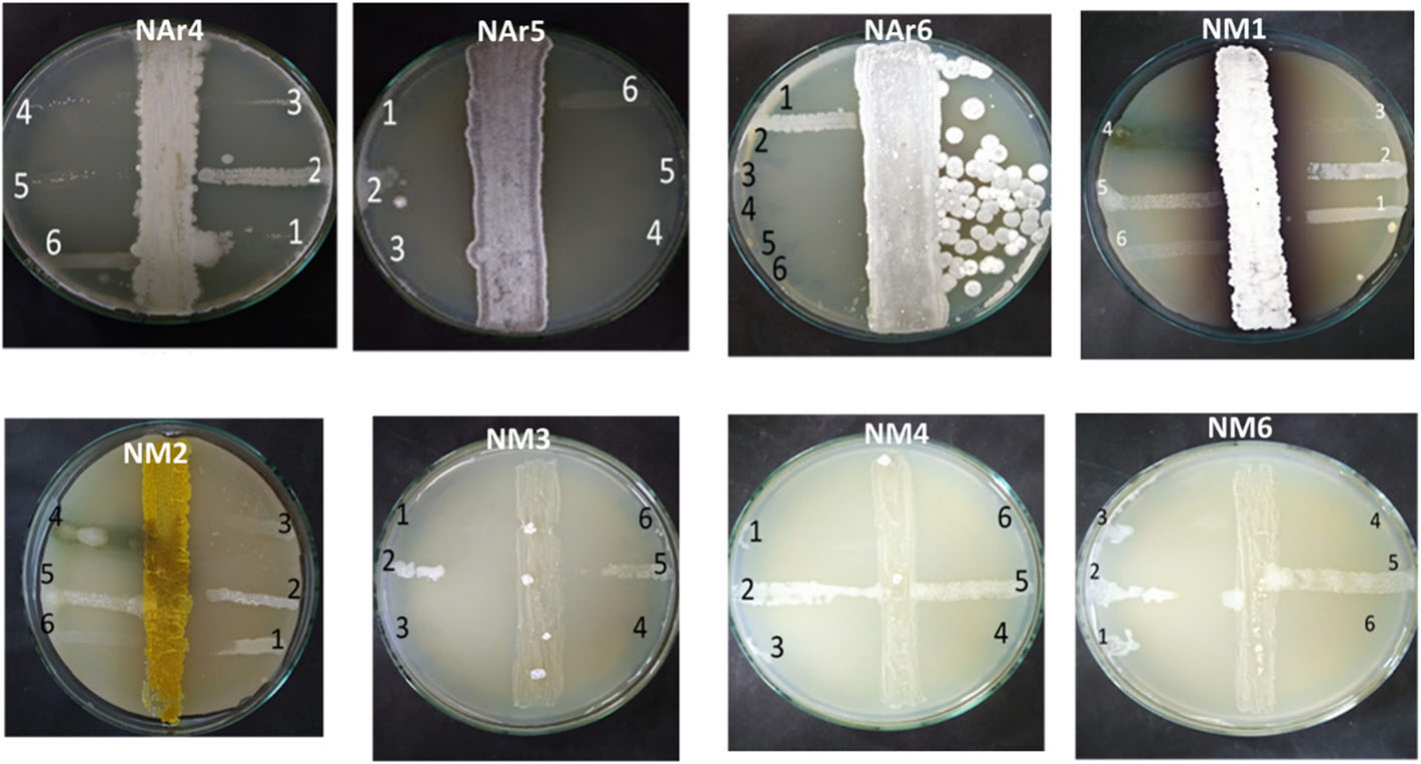
Screening of antibacterial activity of the isolates obtained using resistance guided isolation process against a set of test bacteria. Streak-plating technique was used to screen the antibacterial activity. The actinomycetes strains to be tested were inoculated on YEGA plates in a single line vertically and incubated for 4 days at 32 °C. On the 4th days of incubation, the freshly prepared and properly diluted (adjusted to 0.5 McFarland Standard) test bacteria [1. *S. brodie*; 2. *B. cereus*; 3. *E. coli*; 4. *Pseudomonas sp*; 5. *S. aureus* (multidrug-resistant) and 6. *Proteus sp.*] were inoculated horizontally starting close to the actinomycetes growth line to the end of the plates. The plates were then incubated for 18 h at 37 °C and the DOI was measured in mm using a ruler

To screen the capability of the isolates obtained from the resistance-guided isolation process using azithromycin and meropenem as a screening filter, the cross-streaking method was applied. We found that all the three isolates obtained from azithromycin selection were active and exhibited moderate to high antibacterial activity (Table 2; Fig. 4). The isolate NAr4 inhibited only the Gram-negative bacteria including *S. brodie*, *E. coli, Pseudomonas* and *Proteus sp.* with a distance of inhibition (DOI) ranging from 20 to 38 mm. Both NAr5 and NAr6 exhibited strong antagonistic activity against almost all test bacteria with the exception that NAr6 could not inhibit *B. cereus* (Table 2; Fig. 4).

**Table 2:** Distance of inhibition (DOI) of the actinomycete isolates against the test bacteria^a^

Isolate ID	Gram-negative bacteria				Gram-positive bacteria	
	***S. brodie***	***E. coli***	***Pseudomonas sp.***	***Proteus sp.***	***B. cereus***	***S. aureus***
NAr4	23	32	32	26	-	-
NAr5	30	30	33	32	20	38
NAr6	20	35	35	24	-	32
NM1	35	10	-	35	40	10
NM2	38	30	30	35	22	16
NM3	35	30	27	32	19	10
NM4	37	30	27	35	15	-
NM6	32	24	30	35	16	-

^a^The isolates NAr4-6 were obtained using azithromycin and the isolates NM1-6 were obtained using meropenem as a screening filter. Only the activities of the active isolates are shown

Among the nine isolates obtained by meropenem selection, five exhibited moderate to high broad-spectrum antibacterial activity (Table 2; Fig. 4). The isolate NM1 which produced diffusible red pigments could not inhibit the growth of multidrug-resistant *Pseudomonas sp*. however, it inhibited *S. brodie*, *Proteus sp.* and *B. cereus* strongly. The antagonictic activities of NM1 against *E. coli* and *S. aureus* were not good. The isolates NM2, NM3, NM4 and NM6 exhibited very strong antagonistic activity against Gram-negative bacteria with moderate activity against Gram-positive bacteria (Table 2; Fig. 4). NAr5 and NAr6 were selected for further characterisation and identification based on on their highest antibacterial activity in preliminary antibacterial screening.

### Biochemical characteristics of NAr5 and NAr6

The biochemical characteristics of NAr5 and NAr6 including salt tolerance, pH tolerance, utilization of carbohydrate sources and cross-resistance to other antibiotics were also studied. It was found that both the strains could tolerate a salt concentration up to 8%, however, they grew well when the NaCl concentration was in the range of 3–5%. A pH range of 5–10 was tested and it was found that both the strains could also tolerate this wide range of pH. They also grew well when different carbohydrate sources were used including lactose, maltose, glucose, sucrose, mannitol, starch, fructose and xylose. Both of the strains could grow well in absence of carbon source (starch) of the SCNA medium supplemented with either meropenem or azithromycin. This suggests that they either utilize environmental CO_2_ or the supplied antibiotic as a source of carbon. When the SCNA medium was depleted with nitrogen source (casein and potassium nitrate), the strains could also grow well, thus, there is a possibility that the strains used the supplied antibiotic as nitrogen source. The strains also exhibited multidrug-resistant phenotypes. Along with azithromycin (the antibiotic used as selection filter for both NAr5 and NAr6), both of the strains could tolerate clindamycin and meropenem. The results of the biochemical test of NAr5 and NAr6 are summarized in Table 3.

**Table 3:** Biochemical characterization or test results of NAr5 and NAr6

Type of Biochemical Test	Supplementation of YEGA media	NAr5	NAr6
Carbohydrate source used	Lactose	+++	+++
	Maltose	++	+++
	Glucose	++	+++
	Sucrose	+++	+++
	Mannitol	++	+++
	Starch	+++	+++
	Fructose	+++	+++
	D-Xylose	+++	+++
Salt tolerance test	2 %	++	++
	3 %	+++	+
	4 %	+++	+
	5 %	+++	+
	6 %	++	+
	7 %	++	+
	8 %	+	+
pH tolerance test	pH 5	+++	+++
	pH 6	+++	+++
	pH 7	+++	+++
	pH 8	+++	+++
	pH 9	+++	+++
	pH 10	+++	+++
Utilization of the supplemented antibiotic as a potential carbon source	Meropenem	+++	+
	Azithromycin	+	+++
Utilization of the supplemented antibiotic as a nitrogen source	Meropenem	+++	-
	Azithromycin	-	+++
Growth on in presence of antibiotics	Clindamycin (0.5 µg/ml)	+++	+++
	Azithromycin (0.5 µg/ml)	+++	+++
	Meropenem (4 µg/ml)	+++	+

The sign ‘+++’ indicates abundant production, ‘++’ indicates moderate production, ‘+’ indicates poor production and ‘-’ indicates not grown

### Identification of NAr5 and NAr6 using 16S rDNA sequencing

**Fig. 5:**
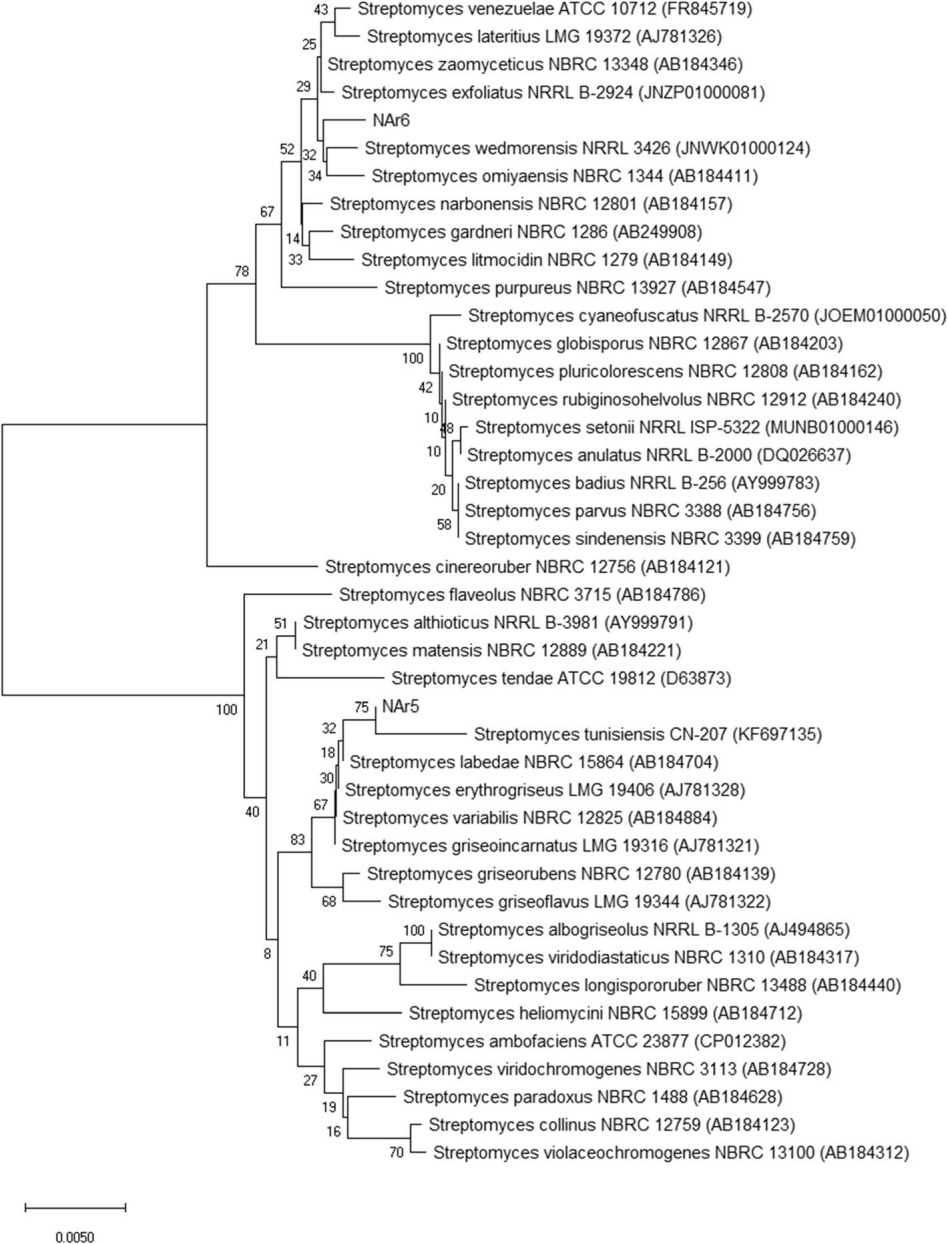
The neighbour-joining tree based on partial 16S rDNA sequences of NAr5 and NAr6. The related taxa were obtained using EzBioCloud database for 16 S rRNA genes [15]. The evolutionary distances were computed using the Maximum Composite Likelihood method [16] and are in the units of the number of base substitutions per site. Evolutionary analyses were conducted in MEGA X [17]

The analysis of the partial 16S rDNA sequences of bothNAr5 and NAr6 suggested that both the strains belonged to the genus *Streptomyces*. The partial 16S rDNA sequence (1283 bp) of the gene of NAr5 shared 100% sequence similarity with five strains of *Streptomyces* including *S. griseoincarnatus* LMG 19316, *S. erythrogriseus* LMG 19406, *S. variabilis* NBRC 12825, *S. griseorubens* NBRC 12780 and *S. labedae* NBRC 15864. Four strains including *S. althioticus* NRRL B-3981 (99.76%), *S. griseoflavus* LMG 19344 (99.69%), *S. tunisiensis* CN-207 (99.69%), *S. matensis* NBRC 12889 (99.69%) showed > 99.5% sequence similarity with the NAr5. In the phylogenetic tree of the 16S rRNA gene sequences of NAr5 with its closely related strains, it formed a separate clade with *S. tunisiensis* CN-207 (Fig.5).

The partial 16S rDNA sequence (1237 bp) of NAr6, another strain obtained from azithromycin selection, shared a maximum 99.76% sequence identity with *S. zaomyceticus* NBRC 13348. Other closely related strains were *S. exfoliates* NRRL B-2924 (99.68%), *S. venezuelae* ATCC 10,712 (99.60%), *S. wedmorensis* NRRL 3426 (99.60%), *S. omiyaensis* NBRC 13449 (99.60%), and *S. lateritius* LMG 19372 (99.60%). In the phylogenetic tree of the 16S rDNA sequences of the closely related strains, NAr6 appeared on a separate branch in the tree, thus it has the potential to be a new species of *Streptomyces, however*, further studies are needed to be performed to confirm this (Fig. 5).

## Discussion

To treat the infections caused by antibiotic-resistant bacteria especially the Gram-negative multi-drug resistant pathogens, new antibiotics are urgently needed. Among the platforms of antibiotic discovery, screening of soil-dwelling microorganisms, popularly known as Waksman platform was once most effective, however, due to the problem of the frequent rediscovery of known compounds, it has now collapsed [3]. The relatively new platforms such as the target-based screening of the large libraries of synthetic compounds also failed, partly due to low penetration of the synthetic compounds into the bacterial cell. This problem can be overcome by using the resistance-guided isolation of antibiotic producers as in this approach small number of strains need to be screened and multiple environments can be sampled [5]. The principle of the resistance-guided isolation method that led to the discovery of pekiskomycin was that the APB possess self-defense against their own product. When the *Streptomyces* isolation agar (SIA) or humic acid vitamin agar is supplemented with an antibiotic, the antibiotic-resistant strains that grow on agar media is likely the producer of antibiotics with similar scaffolds [4, 5].

Here in this study, we performed a resistance-guided screening of antibiotic-producing bacteria present in the river-bed sediments of Padma and Kapotakkho rivers of Bangladesh. Although several studies have been carried out previously to isolate the actinomycetes capable of producing bioactive compounds from soils and marine sediments from Bangladesh [18–20], no studies have been performed using the river bed sediments. We observed that supplementation of starch-casein-nitrate agar (SCNA) selective media with either azithromycin (a semisynthetic macrolide antibiotic) or meropenem (a semisynthetic carbapenem) decreased the number of contaminating bacteria on the agar plates and thus, the total number of colonies appeared on the plates decreased significantly compared to the control plates where no antibiotics were supplemented. We have also seen that, when the concentration of the antibiotic is increased in the isolation media, there is a proportionate decrease of the colony counts on the plates. So, some of the actinomycetes strains that appear on a lower concentration did not appear on a higher concentration of antibiotics. At very high concentrations, in some cases, no actinomycetes like colonies appeared, and only some fungal colonies grew.

The previous studies on supplementation of antibiotics for selection also produced similar results. For instance, when rifampicin was supplemented to the SCNA media to isolate actinomycetes from marine sediments, the number of CFU decreased with an increase in the concentration of rifampicin on the plates [11]. Similarly, when vancomycin was supplemented to the isolation media, about 96% of the actinomycete strains that do not produce glycopeptide antibiotic (GPA), did not grow on the plates which allowed exclusive growth of resistant isolates that produce GPA. It also provided the opportunity to grow the slow-growing strains as well as the less-abundant actinomycetes [4].

Using the resistance-based isolation approach we found that 100% (3 out of 3) of the azithromycin resistant isolates exhibited antibacterial activities. Two of them (NAr5 and NAr6) exhibited broad-spectrum activity and one (NAr4) was active against the gram-negative bacteria only. However, in the case of meropenem-resistant actinomycete isolates, approximately 56% (5 out of 9) of the isolates exhibited antibacterial activity during the initial screening. The percentage of actinomycete isolates that exhibit antibacterial activity towards at least one type of bacteria (Gram-positive or Gram-negative) varies depending on the source. In a previous study conducted in our labs using the traditional Waksman approach, we found that about 54% of the actinomycetes obtained from various kinds of surface soils produced antibacterial compounds [18]. In another study of screening for actinomycetes producing antibacterial compounds using the soils of Sundarban, Bangladesh, about 36% of the actinomycete isolate were active against the indicator bacteria [20]. In another study using the different kinds of samples (water and sediment) from both marine and freshwater sources, 23% of the isolates (27 out of 119) exhibited antibacterial activity [21]. During a screen of the soil samples of the West of Iran, the percentage of active isolates was only 13.30% [22]. Thus, compared to these previous studies, screening of the antibiotic-producing actinomycetes using meropenem as a screening filter offered an increased output.

Azithromycin is a semisynthetic broad-spectrum antibiotic of 15-membered-ring macrolide that works by inhibiting the protein synthesis of bacteria. Similar to erythromycin, it binds with the 50S large ribosomal subunit and inhibits the growth of the nascent polypeptide chain [23]. The mechanisms of resistance to azithromycin as well as other macrolides in the clinical pathogens include: i) target modifications by rRNA methylases or by mutations, ii) inactivation by esterases or by phosphotransferase and iii) macrolide-efflux pumps [24–26]. In the antibiotic-producing soil bacteria, the mechanisms of macrolide resistance include ribosomal modification [27], efflux-pump [28] and inactivation by glycosyltransferase-mediated glycosylation [29]. The mechanism of resistance by glycosylation has only been found in APB as a self-resistance mechanism and has not been reported in the clinical pathogens. For instance, oleandomycin producing *Streptomyces antibioticus* confers self-resistance by 2’-glucosylation of the antibiotic [29]. Macrolide resistance in *Streptomyces lividans* also occurs due to glycosylation [30]. Considering these examples of macrolide resistance mechanisms in both clinical pathogens and the antibiotic producing soil bacteria, it can be suggested that azithromycin-resistant isolates of our study used the target modification or the efflux pumps as their resistance mechanisms.

Meropenem is a member of carbapenems which possess broad-spectrum antibacterial activity, having a distinctive structure of a carbapenem coupled to a β-lactam ring which exhibits defence against a range of β lactamases such as Metallo-β-lactamase (MBL) as well as extended-spectrum β-lactamases [31]. Meropenem possesses a common beta-lactam ring-like other β-lactam antibiotics, and act similarly by binding to and inactivating the penicillin-binding proteins (PBPs), which are responsible for the development of the bacterial cell wall [32]. The most common mechanisms of meropenem resistance, as well as other carbapenems in the clinical pathogens, include: (i) enzymatic inactivation by β-lactam-hydrolyzing enzymes, (ii) target site mutation (mutation-derived from changes of their PBPs), and (iii) overexpression of efflux pumps [33]. Antibiotic producing soil bacteria develop resistance to meropenem as well as other carbapenems through (i) producing carbapenem-hydrolyzing enzymes (carbapenemases) (ii) decreasing outer membrane permeability, and (iii) increasing efflux mechanism [34].

The analysis of 16S rDNA sequences of NAr5 and NAr6 revealed that both belong to the genus *Streptomyces*. The cultural, physiological, and morphological characteristics of the isolates also matched with the genus *Streptomyces.* In the phylogenetic tree constructed with the 16S rDNA sequences, the NAr5 appeared on the same branch with *S. tunisiensis* CN-207 (Fig. 5). The *S. tunisiensis* CN-207 was isolated from Tunisian soils and was found to exhibit strong antibacterial activities against both gram-positive and gram-negative bacteria [35], however, the compound(s) responsible for the antibacterial activity has not been identified.

The isolate NAr6 appeared on a separate branch in the phylogenetic tree (Fig. 5). It exhibited the highest 16S rDNA sequence identity with *S. zaomyceticus* NBRC 13,348. A strain of *S. zaomyceticus* was isolated from soil and it exhibited activity against both methicillin-resistant *Staphylococcus aureus* (MRSA) and methicillin-sensitive *Staphylococcus aureus* (MSSA)[36]. Another strain of *S. zaomyceticus* produces an antibiotic zaomycin [37]. Zaomycin is an antibiotic related to amphomycin, a lipopeptide antibiotic produced by different species of *Streptomyces* and *Actinoplanes* [38]. Further works are being carried out in our laboratory to identify the compounds that are produced by NAr5 and NAr6.

This study has further provided evidence that the river sediments are a very potential source of APB and more investigations may lead to the isolation of novel APB and potentially new antibiotics having activity against clinically important pathogens. We have also confirmed the advantage of using a self-resistance mechanism to identify the antibiotic producers by eliminating the contaminating bacteria present in the samples, thus facilitates the growth of difficult to grow actinomycetes on the isolation plates. The isolates resistant to respective antibiotics used for selection have the potential to produce antibacterial compounds of a similar scaffold.

## Methods

### Source of chemicals, reagents, antibiotics, and solvents

Starch and agar powder were purchased from Merck, Germany; potassium nitrate (KNO3), potassium phosphate dibasic (K_2_HPO_4_), calcium carbonate (CaCO_3_), magnesium sulfate (MgSO_4_) and sodium chloride (NaCl) from Sigma-Aldrich. Yeast extract was purchased from HiMedia, India. The antibiotics were obtained from different local pharmaceutical companies as a donation. The antibiotic susceptibility disks were purchased from Liolfilchem, Italy. Solvents were purchased from Daejung Chemical, South Korea.

### Bacterial strains used to investigate the antibacterial activity

The test bacteria used to study the antibacterial activity of the isolated soil actinomycetes were donated from the Department of Microbiology, Rajshahi Medical College, Rajshahi, Bangladesh. Among the six test bacteria, two were gram-positive (*Bacillus cereus* and *Staphylococcus aureus*) and four were gram-negative (*Shigella brodie, Escherichia coli, Pseudomonas sp*, and *Proteus sp*). The test bacteria were maintained and grown in Nutrient Agar medium (Hi Media, India) and preserved both in agar slant at 4 °C and 20% v/v glycerol at -20 °C.

### Sampling sites and collection of river sediments

The sediment samples were collected from two rivers of Bangladesh, Padma river, Rajshahi (24.3614252 N,88.5991637E) and Kapotakha river, Jashore (23.1061737 N,89.0937548E). Soil sediments were collected from a various depth of the rivers (2–4 m). A clean bamboo was dipped into the river and the sediment got stuck inside the bamboo hole was collected using a sterile spatula and transferred into sterile conical tubes and carried to the lab for analysis.

### Preparation of isolation media supplemented with azithromycin and meropenem

The Starch-casein-nitrate-agar (SCNA) media (composition: soluble starch: 10 g, K_2_HPO_4_: 2 g, KNO_3_: 2 g, casein: 0.3 g, MgSO_4_.7H_2_O: 0.05 g, CaCO_3_: 0.02 g, FeSO_4_.7H_2_O: 0.01 g, agar: 15 g, and filtered seawater: 1000 ml and pH: 7.0 ± 0.1). The azithromycin and meropenem powders were dissolved into sterile water to make a stock concentration of 1 µg/mL and filter sterilized using 0.22 µM syringe filters (Millex, Sigma). An appropriate volume of antibiotics to make concentration equal to and higher than MIC of the antibiotics was added to the media cooled to 55°C to prepare the SCNA plates. To prepare SCNA plates with 2, 4 and 16 µg/mL of azithromycin, 2, 4 and 15 mL of azithromycin stock was added to 1 L of molten agar, respectively (MIC of azithromycin = 2 µg/mL). Accordingly, to prepare SCNA plates with 4, 16 and 64 µg/mL of meropenem, 4, 16 and 64 mL (MIC of meropenem ≥ 4 µg/mL) of the stock meropenem solution was added to 1 L molten agar, respectively. SCNA plates not supplemented with azithromycin were used as negative controls. The plates were prepared freshly.

### Plating of serially diluted soil sediments on azithromycin supplemented SCNA plates

1 g soil was diluted in 10 ml of sterile saline solution (0.9% NaCl). Three different dilutions (1:10, 1:100 and 1:1000) were prepared using sterile saline solutions in a total volume of 10 ml. 100 µL samples of each dilution were plated to azithromycin or meropenem supplemented SCNA plates separately containing three different concentrations of antibiotics and to the control plates. The plates were incubated at 32°C up to 14 days to allow the bacteria to grow.

### Colony count for each dilution plate and isolation of pure culture of the actinomycetes

Colonies having actinomycete like morphologies (round, small, opaque, tough, leathery, velvety, frequently pigmented colonies with filamentous growth) were then counted and recorded. Colonies having different colours and appearances were randomly picked using sterile toothpicks and inoculated in fresh SCNA plates carrying the same concentration of antibiotics from where the isolates were picked. The pure isolates were transferred to fresh antibiotic supplemented SCNA plates for at least two times to confirm their purity.

### Morphological characteristics of the isolates

All morphological characteristics of the isolates were studied on yeast extract glucose agar (YEGA) plates (composition: glucose: 10 g, yeast extract: 1 g, potassium nitrate: 1 g, potassium monohydrogen phosphate: 0.1 g, agar: 15 g, distilled water q.s to 1000 mL). The growth, aerial spore-mass colour, substrate mycelium colour and pigment production were observed for phenotypic grouping of the isolates. The plates were examined by naked eyes and a National Bureau of Standards Colour Chart was used to determine the colour of the substrate mycelia and aerial mycelia [39]. The pure colonies were inspected under a light microscope for the structure of spore chains and their Gram-staining characteristics.

### Screening of the isolates for antibacterial activity

The antibacterial activities of the drug-resistant isolates were tested using the cross-streaking plating technique as described previously [18]. Briefly, the pure isolates were streaked individually on YEGA agar plates in a single line from one end of the plate to other. The plates were then incubated at 32 °C for 4 days to allow the isolates to secrete antibiotics into the medium. After the end of the incubation period, the freshly grown test bacteria at their log phase was adjusted to 0.5 McFarland standard solutions and were cross streaked along the line of the fully grown isolates. The distances in millimeter (mm) to which the growth of the test bacteria was inhibited along the line of the actinomycetes growth line was measured.

### Biochemical characteristics of the isolates

The biochemical tests included utilization of different carbon sources, salt tolerance, pH tolerance, utilization of the supplemented antibiotics as sole carbon or nitrogen source as well as cross-resistance to other antibiotics. To determine if the isolates could grow without supplemented carbon sources, starch (carbon source) was not added to the SCNA media and the media was inoculated with the freshly grown isolates in spots. Similarly, to determine if the isolates could grow without supplemented nitrogen source, they were grown on the media deficient of casein and potassium nitrate.

### Isolation of genomic DNA

A single pure colony of the isolates was inoculated into YEGA media and grown at 32 °C for 3 days. To check the purity of the broth culture on 3 days incubation, 100 µL of the culture was spread onto the YEGA agar plate and checked if any contaminant bacteria grow on the plates. 1.5 ml of the 3-days-old broth culture of the isolates was used to isolate the genomic DNAs using the Wizard Genomic DNA Purification Kit (Promega) following the manufacturer’s instructions for Gram-positive bacteria. The purified genomic DNAs were checked on 1% agarose gel to check the purity.

### PCR amplification, sequencing of 16S rDNA gene

PCR amplification of the 16S rDNA from each sample was performed using Hot Start Green Master MixM7432 (Promega, USA). A 20 µl PCR reaction mix contained 10 µl Master Mix (10X), 1 µl of gDNA (concentration 25–65 ng/ul), 1 µl of both primers 27F (5’- AGAGTTTGATCMTGGCTCAG-3′) and 1492R (5’-CGGTTACCTTGTTACGAC TT-3′) and 7 µl molecular grade H_2_O. The PCR conditions were as follows: initial denaturation for 5 min at 95°C, then 35 cycles of denaturation for 30 s at 95 °C, annealing for 30 s at 48 °C, an extension for 1.5 min at 72°C and final extension for 5 min at 72 °C. The quality of the PCR products was checked in 1% (w/v) agarose gel (V3125, Promega, USA) and visualized in a gel documentation system (Alpha Imager, USA). The amplified DNA fragments were purified using the PCR Clean-Up System (A9281, Promega, USA) and sequenced using Sanger Sequencing.

### Phylogenetic analysis of the partial 16S rDNA sequences of NAr5 and NAr6

The homologous sequences of the partial 16S rDNA sequences were obtained using EZBiocloud.net[15]. The homologous sequences were then aligned using Clustal X [40]. The phylogenetic analysis of the 16S rDNA sequences of NAr5 and NAr6 were performed using the MEGAX software (http://www.megasoftware.net/) [17]. The neighbor-Joining method [41] was applied to construct the phylogenetic tree.

### GenBank Accession Numbers

The partial 16S rDNA sequences of NAr5 and NAr6 were submitted to GenBank with the accession numbers MT483566 and MT483567, respectively.

## Data Availability

The 16 S rDNA sequences of the strains NAr5 and NAr6 have been submitted to GenBank and they are now publicly available with the accession numbers of MT483566 and MT483567, respectively.
